# The prevalence of awake bruxism and sleep bruxism in the Dutch adolescent population

**DOI:** 10.1111/joor.13117

**Published:** 2020-11-08

**Authors:** Peter Wetselaar, Erik J. H. Vermaire, Frank Lobbezoo, Annemarie A. Schuller

**Affiliations:** ^1^ Department of Orofacial Pain and Dysfunction Academic Centre for Dentistry Amsterdam (ACTA) University of Amsterdam and Vrije Universiteit Amsterdam Amsterdam The Netherlands; ^2^ TNO Child Health Leiden The Netherlands; ^3^ Center for Dentistry and Oral Hygiene University Medical Center Groningen Groningen The Netherlands

**Keywords:** adolescent population, awake bruxism, gender, prevalence, sleep bruxism, socio‐economic status

## Abstract

**Objectives:**

This study aimed to assess the prevalence of awake bruxism and sleep bruxism in the Dutch adolescent population.

**Materials and Methods:**

As part of a large epidemiologic survey on oral health of the general Dutch adolescent population in 2017, a total of 920 subjects were asked about their bruxism behaviour during daytime and during sleep. The collected data were subjected to stratified analysis by two age groups (for 17 and 23 years, respectively), gender and socio‐economic status.

**Results:**

A prevalence of 4.1% and 4.2% was found for awake bruxism and of 7.6% and 13.2% for sleep bruxism. Women reported awake bruxism more often than men in the 17‐year‐old age group (5.0% and 3.2%, respectively), while in the 23‐year‐old age group it was the other way around (4.0% and 4.4%, respectively). Regarding sleep bruxism, women reported higher percentages than men in both age groups (7.8% versus 7.5% and 14.9% versus 11.5%, respectively). Concerning socio‐economic status (SES), awake bruxism was more often found in high SES groups (4.6% versus 3.7% and 4.9% versus 4.0% in both age groups, respectively) as well as for sleep bruxism in the 23‐year‐old group (16.5% versus 8.6%). In the 17‐year‐old group, sleep bruxism was more often reported in the low SES group (9.7% versus 5.3%).

**Conclusions:**

Sleep bruxism is a common condition in the Dutch adolescent population, while awake bruxism is rarer.

**Clinical relevance:**

Dental caregivers can use this information when negative healthcare outcomes are present amongst adolescents.

## 1 INTRODUCTION

In 2018, a group of experts redefined bruxism regarding both circadian manifestations (ie, awake and sleep bruxism) and reformulated the conditions' diagnostic grading.^1^ It was stated that awake bruxism and sleep bruxism are considered to be different behaviours observed during wakefulness and during sleep. The subsequent proposed definitions were ‘sleep and awake bruxism are masticatory muscle activities that occur during sleep (characterised as rhythmic or non‐rhythmic) and wakefulness (characterised by repetitive or sustained tooth contact and/or by bracing or thrusting of the mandible), respectively'. Furthermore, the statement was made that in otherwise healthy individuals, bruxism should not be considered as a disorder, but rather as a behaviour that can be a risk (and/or protective) factor for certain clinical consequences. It is described that bruxism can be a risk factor with possible negative oral health outcomes, such as severe masticatory muscle pain or temporomandibular joint pain, extreme mechanical tooth wear, cracked teeth and/or prosthodontic complications. For the assessment of bruxism, both non‐instrumental approaches (notably self‐report) and instrumental approaches (notably electromyography) can be employed, leading to three stages of the likelihood to correctly diagnose awake bruxism and sleep bruxism, namely 'possible' (notably self‐report), 'probable' (with clinical inspection) and 'definite' (notably electromyography). Finally, it was advised that standard cut‐off points for establishing the presence or absence of bruxism should not be used in otherwise healthy individuals, but that bruxism‐related masticatory muscle activities should be assessed in the behaviour's continuum.^1^

A review regarding prevalence data amongst adult populations shows that studies on bruxism are scarce and have a wide range from 8% to 31.4%.^2^ The same applies to childhood or adolescent populations, where a range of 3.5% to 40.6% was reported.^3^, ^4^ This variety is caused by several factors, namely the fact that some researchers did not specify the type of bruxism (awake/sleep), let alone its likelihood (possible/probable/definite). For the assessment of ‘possible’ awake or sleep bruxism, no consensus was reached on which questions and/or questionnaires should be used to set the diagnosis. Nevertheless, chairside questions and/or questionnaires are tools that can be applied relatively easily to larger groups of individuals. However, it is not always clear whether obtained data form studies can be plainly extrapolated to the general population. Furthermore, the age range of what is considered adolescence differs. Recently, it was suggested that rather than age 10‐19 years, a definition of 10‐24 years corresponds more closely to adolescent growth and popular understandings of this stage of life.^5^

In their review, Barbosa et al concluded that the prevalence of sleep bruxism in childhood and adolescence ranges between 7.0% and 15.1%, with girls apparently more frequently affected.^3^ Amongst the aetiological factors of bruxism that are mentioned in a review,^6^ it was stated that in younger children also the immaturity of the masticatory neuromuscular system may play a role.^7^ Manfredini et al reported an even higher variability in the studies they included in their review, ranging from 3.5% to 40.6%, with a decrease with age and no gender difference.^4^ The authors concluded that this variability was due to the different age groups being studied.^4^ Lavigne & Montplaisir showed a decrease in the prevalence of possible sleep bruxism amongst Canadians in relation to age, from 13% in the age range 18‐29 year to 3% at the age of 70 years without a gender difference.^8^ After the above‐mentioned reviews,^3^, ^4^ eight surveys in childhood and adolescent populations were published.^9^, ^10^, ^11^, ^12^, ^13^, ^14^, ^15^, ^16^

The aim of this study was to assess the prevalence of possible awake bruxism and possible sleep bruxism in the Dutch adolescent population in different age groups, for both genders and for different socio‐economic status, using a representative sample.

## 2 MATERIALS AND METHODS

### 2.1 Study sample and recruitment

**Figure 1:**
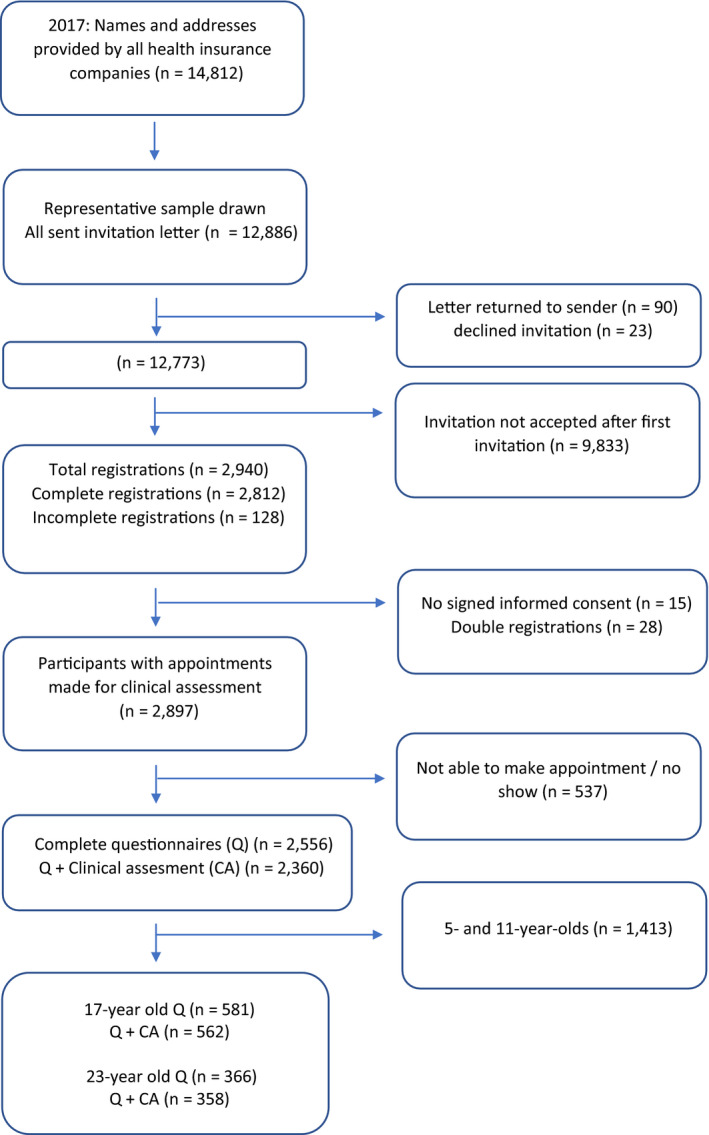
Flow chart of inclusion of participants

Data from 17‐ and 23‐year‐old inhabitants of four medium‐sized cities in The Netherlands were collected in 2017 as part of a large epidemiologic survey of oral health and preventive behaviour amongst Dutch children and adolescents.^17^, ^18^ These four cities (Alphen aan den Rijn, Breda, Gouda and ‘s‐Hertogenbosch) can—together—be considered to be representative of the general Dutch population in terms of sociodemographic indicators, like distribution by age, educational level, migration background, household and marital status.^18^, ^19^ Under authority of the National Health Care Institute Netherlands (ZIN), names and addresses of all eligible participants, born in 1994 and 2000, were collected. Figure 1 shows the flowchart of inclusion of the participants. Informed consent for participating in the clinical examination was signed. Persons who did not respond were contacted face to face by trained interviewers who emphasised the importance of the study. In case of non‐contact, the interviewer returned up to a maximum of three contact attempts. Individuals who refused participation were asked to fill out a non‐response questionnaire, with questions about gender, socio‐economic status (SES) and oral health behaviour. The power calculation upon the primary outcome of the original study (caries experience) indicated that 525 17‐year‐olds and 350 23‐year‐olds had to be included in the clinical examination. Recruitment of new participants stopped when these numbers were reached. The study was judged by the Central Committee on Research Involving Human Subjects (CCMO) not to fall under the provisions of the Medical Research Involving Human Subjects Act. The study met all requirements of the Personal Data Protection Act (approval number m1638552).

### 2.2 Procedure

All participants completed a self‐administered questionnaire providing details of their sociodemographics, dietary and oral hygiene behaviour and their self‐perceived dental status. Socio‐economic status (SES) was defined by the level of education. Level of education was divided into low and high, based upon the intellectual challenges of the Dutch education system. High education was defined as at least higher general secondary education. All other education was defined as low education. Prior to the clinical assessment, all participants were interviewed about their awake bruxism and sleep bruxism behaviour. These questions were asked by experienced, trained and calibrated dentists (n = 8), who also participated in previous surveys.^17^, ^18^, ^21^ For the purpose of this study, the following two questions about bruxism, modified from Van der Meulen et al,^20^ were asked: 1. do you grind your teeth or do you clench your jaws while you are awake? and 2. did someone mention or are you aware yourself that you grind your teeth or clench your jaws during sleep? Regarding these questions, participants were offered three possible answers: yes/ no/ I don't know. For data analyses, the answers were dichotomised into ‘yes’ and ‘no/don't know’.

### 2.3 Statistical analysis

Data were used from participants whose clinical data had been collected and who had answered the question about SES (17‐year‐olds n = 562; 23‐year‐olds n = 358). To identify whether the people that had declined to participate differed from the participants, a modest non‐respondents survey was executed, describing what the main reasons had been for refusing to participate in previous studies. Individuals with high SES were more willing to participate than individuals with low SES. Females were more willing to participate in the study than males (Table 1). To assess population estimates, we therefore weighted our results to population references on SES and gender according to Statistics Netherlands (Table 2). We re‐balanced the sample by weighting by gender, SES and age in order to give a more accurate reflection of the Dutch population. Care must be taken to watch the minimum and maximum balancing weights and the ratio between them, in order to avoid creating a situation in which a group of individuals were counted as many individuals and another group were counted as only a few individuals. We therefore also presented our results stratified by gender, SES and age in order to get more insight into the unweighted results and the effect of these factors on awake and sleep bruxism. The models show that the effects of age, gender and SES are not statistically significant for awake bruxism, but significant effects are shown for age and the interaction between SES and age for sleep bruxism. Therefore, the effect of weighting was small for awake bruxism. However, for sleep bruxism, the importance of weighting was apparent. Without weighting, the proportion of sleep bruxism would have been too low in the 17‐year‐old group and too high in the 23‐year‐old group to properly represent the Dutch population. We expect that weighting reduces most of this bias. A logistic regression model was used with sleep and awake bruxism as dependent variables, and age, SES and gender as independent variables. Model 1 estimated the main effects; Model 2 added two interaction terms to the model. All analyses were performed using IBM SPSS Statistics 25 software (IBM Corp., Armonk, NY, USA).

**Table 1:** Distribution according to gender and socio‐economic status (SES)

		17‐y‐olds n = 562	23‐y‐olds n = 358
Gender	% Male	42 (50)	33 (50)
	% Female	58 (50)	67 (50)
SES	% Low	39 (53)	25 (42)
	% High	61 (47)	75 (58)

Note

In parentheses the distribution from national databases Statistics Netherlands.

**Table 2:** Weighted frequency distributions of awake bruxism and sleep bruxism according to age, gender, and socio‐economic status (SES), weighted

	n	Total % yes	Female % yes	Male % yes	Low SES % yes	High SES % yes
17‐y‐olds						
Awake bruxism	562	4.1	5.0	3.2	3.7	4.6
Sleep bruxism	562	7.6	7.8	7.5	9.7	5.3
23‐y‐old						
Awake bruxism	356	4.2	4.0	4.4	4.0	4.9
Sleep bruxism	357	13.2	14.9	11.5	8.6	16.5

## 3 RESULTS

The non‐respondents survey (17‐year‐olds n = 171; 23‐year‐olds n = 90) showed that 89% of the 17‐year‐olds and 86% of the 23‐year‐olds did not participate in the survey due to lack of time, lack of interest or because they had moved. Also mentioned were dental anxiety (both age groups 7%) and other reasons (respectively, 8% and 7% for 17‐ and 23‐year‐olds).

Table 2 shows the population estimates of prevalence of possible awake bruxism and possible sleep bruxism by SES and gender in 17‐ and 23‐year‐olds. The overall weighted population estimates for awake bruxism were 4.1% in 17‐year‐olds and 4.2% in 23‐year‐olds. The overall weighted population estimates for sleep bruxism were 7.6% in 17‐year‐olds and 13.2% in 23‐year‐olds.

Table 3 shows the results of the logistic regression models with awake bruxism and sleep bruxism as dependent variables, and age, gender and SES as independent variables (model 1), and the interactions between age and gender, and age and SES (model 2). A clear age effect on sleep bruxism was shown in model 1, in which the odds of having sleep bruxism was 2.3 times higher in 23‐year‐olds than in 17‐year‐olds. Model 2 showed that this was mainly due to the high SES group; the odds for having sleep bruxism were 3.0 higher in 23‐year‐olds with a high SES than in 17‐year‐olds with a high SES.

**Table 3:** Logistic regression analyses with awake bruxism and sleep bruxism as dependent variables

	Model 1			Model 2		
	Adj. OR	95% CI	*P*	Adj. OR	95% CI	*P*
Awake bruxism						
Age (ref = 17 y)	0.92	[0.48; 1.76]		1.71	[0.40; 7.32]	
Gender (ref = male)	1.12	[0.59; 2.15]		1.38	[0.60; 3.14]	
SES (ref = low)	1.29	[0.65; 2.58]		1.48	[0.63; 3.47]	
SES × age				0.69	[0.16; 2.90]	
Gender × age				0.59	[0.16; 2.23]	
Constant	0.04			0.03		
Sleep bruxism						
Age (ref = 17 y)	2.33	[1.48; 3.69]	*	1.13	[0.40; 3.22]	
Gender (ref = male)	1.15	[0.72; 1.86]		1.14	[0.57; 2.28]	
SES (ref = low)	1.03	[0.63; 1.68]		0.63	[0.32; 1.24]	
SES × age				2.95	[1.03; 8.41]	*
Gender × age				0.97	[0.38; 2.53]	
Constant	0.06			0.08		

Abbreviation: SES, socio‐economic status.

* *P* < .05.

## 4 DISCUSSION

The aim of this study was to assess the prevalence of possible awake bruxism and possible sleep bruxism in the Dutch adolescent population. Sleep bruxism appeared to be a common condition, while awake bruxism is rarer. The results will be discussed regarding the following aspects: (a) sample, sample size, age pattern; (b) prevalence, awake bruxism versus sleep bruxism; (c) gender, SES; and (d) questioning.

### 4.1 Sample, sample size, age pattern

First of all, it is important to note that different definitions of adolescence are used in the available published papers. We followed the suggestion of Sawyer and coauthors that adolescence is the stage of life between 10 and 24 years. In their review paper regarding the prevalence of bruxism in children, Manfredini and coauthors stated that the sample sizes of the included papers differed considerably.^4^ Since publication of this review, eight additional studies were performed in adolescent populations worldwide. These studies were performed in Canada,^10^ the Netherlands,^11^ Japan,^9^ Israel ^12^, ^16^ and Brazil.^13^, ^14^, ^15^ The Canadian study included 604 adolescents of 7‐17 years old, the Japanese study included 99,416 adolescents of 12‐18 years old, The Dutch study included 4,235 adolescents of 12‐18 years old, the Israeli studies included 1,000 adolescents of 12‐18 years old and 1,019 adolescents of 14‐18 years old, respectively, and finally the Brazilian studies included 253 adolescents of 18‐30 years old, 231 adolescents of 12 years old and 594 adolescents of 11‐14 years old, respectively. Except for the Japanese study, the recruited samples could not be considered as representative for the populations. The present study used a weighting procedure of the study sample, calculating population estimates. Therefore, the presented results can be regarded representative for the whole Dutch population. Eight of the above‐mentioned studies presented their results on aggregate, resulting that no age pattern could be revealed. Only the Israeli study reported the results in two age groups (12‐15 years and 16‐18 years).^12^ By presenting data in two different age groups, the present study facilitates age pattern analyses. The results of this study showed an increase in the prevalence of sleep bruxism with increasing age. The age difference can be explained by an increase in masticatory muscle activity, or by the fact that adolescents of that age often have a bed partner who can report their grinding during the night, or both. Of course, this is a shortcoming of self‐report as mentioned earlier.

### 4.2 Prevalence, awake bruxism versus sleep bruxism

In our survey, it was revealed that possible awake bruxism is a rarer condition (4.1% and 4.2% in 17 year old and 23‐year old, respectively) than possible sleep bruxism (7.6% and 13.2% in 17 year old and 23‐year old, respectively). A third strength of this study was that the two circadian conditions were assessed separately. In some of the other surveys in which awake bruxism and sleep bruxism were assessed separately, this same trend was revealed, although with different percentages. These percentages were for probable awake bruxism and sleep bruxism in Canada 12.4% and 15.0%, respectively,^10^ and for probable awake bruxism and sleep bruxism in the Netherlands 8.7% and 14.8%, respectively.^11^ In the two Israeli surveys, it was the other way around: a higher prevalence of probable awake bruxism as compared to sleep bruxism was found, viz., 19.2% and 9.2%^12^ and 34.5% and 14.8%,^16^ respectively. One Brazilian study assessed probable awake bruxism and sleep bruxism together, with a percentage of 31.6%.^13^ The three remaining studies only assessed sleep bruxism, with percentages of 2.3% possible sleep bruxism,^9^ 16.9% probable sleep bruxism^14^ and 22.2% probable sleep bruxism,^15^ respectively. As described in the earlier mentioned review,^4^ a huge prevalence range of 3.5%‐40.6% exists between studies. The Japanese study even revealed a prevalence beyond this described range, all the other seven studies were within these range. More studies are needed to construct a framework of ‘normality’ figures of awake bruxism and sleep bruxism. Furthermore, as mentioned in the introduction, future studies need to assess awake bruxism and sleep bruxism separately, since they are considered as two separate behaviours.

### 4.3 Gender and SES

Logistic regression analyses showed that the risk of sleep bruxism increased with age, and more frequently in the high SES group. Gender did not add any statistically significant effects. This is in line with three other studies also reporting equal prevalence for boys/men and girls/women.^10^, ^11^, ^12^ Only one Israeli study reported that girls/women showed more bruxism than boys/men.^16^ The gender difference in this Israeli study revealing higher prevalence in girls/women was, however, not in line with the survey of our Dutch colleagues,^11^ the Japanese survey^9^ and the other Israel studies.^16^ One of the Brazilian studies reported a higher prevalence amongst boys/men.^15^ No gender difference was found in the four other studies.^10^, ^12^, ^13^, ^14^ Concerning SES, our results were partly in line with the only other study that assessed the effect of SES on awake bruxism and sleep bruxism, finding no difference for both activities.^11^

### 4.4 Questioning

Forty per cent of the 23‐year‐olds lived together with a partner. The individuals with partner did not report sleep bruxism more often than those living without a partner, leading to the conclusion that self‐report of bruxism appears to be reasonable valid. It is important to continue to realise that self‐report has its limitations and shortcomings in general, and of course in bruxism research as well. Therefore, further research is needed to improve the non‐instrumental assessment tools for bruxism. Nevertheless, for now, self‐reported assessment of sleep and awake bruxism continues to be the primary tool used in bruxism research and clinical practice.^1^ This is the reason that we used this approach in our survey as well. It is clear that for the above‐mentioned reasons, the results of this survey must therefore be interpreted with caution.

In most publications on adult awake and sleep bruxism, no uniformity on the posed questions and answering options could be retrieved.^21^ Also in the above‐mentioned papers on awake and sleep bruxism in adolescence, different questions with different answering options, regarding the muscle activities were used. Besides the difference in questions, also different answering options were used in different studies. Six studies used the answering option ‘yes’ or ‘no’, or the answers were dichotomised.^9^, ^10^, ^11^, ^12^, ^14^, ^16^ Two studies did not mention the exact question in their material and methods.^13^, ^15^ In the present study, the questions were asked to the adolescents themselves. Five studies posed the question to the adolescents themselves as well,^9^, ^11^, ^12^, ^13^, ^16^ and three other studies asked the parents.^10^, ^14^, ^15^ To make comparison possible, this study used the same procedure in questioning and answering as our Dutch colleagues,^11^ and we did in our survey in the Dutch adult population.^21^ To improve comparability between countries in future adolescent studies, consensus is needed regarding the exact questions and answering options, whom to ask in which age categories (participants or parents), as the way of posing questions may definitely influence outcomes.^22^

## 5 CONCLUSION

The results of assessing possible awake bruxism and possible sleep bruxism, being part of a large epidemiologic survey on oral health of the general Dutch adolescent population, revealed a prevalence of 4.1%‐4.2% for awake bruxism and of 7.6%‐13.2% for sleep bruxism. Awake bruxism is stable over the age groups, while sleep bruxism increases with age (statistically significant), and then mostly in high SES groups. Sleep bruxism is a common condition in the Dutch adolescent population, while awake bruxism is rarer. Knowing that sleep bruxism is a common condition amongst Dutch adolescents, can help dental caregivers when confronted with negative healthcare outcomes.

## CONFLICT OF INTEREST

The authors declare no conflicts of interest.

## AUTHOR CONTRIBUTIONS

PW, JV, FL, and AS conceived and designed the study. JV and AS performed the clinical data collection PW, JV, and AS analysed the data. PW, JV, and AS wrote the paper. FL subjected the main intellectual content of the manuscript to critical review. All authors read and approved the final manuscript.

### Peer Review

The peer review history for this article is available at https://publons.com/publon/10.1111/joor.13117.
